# Topical nitroglycerin to detect reversible microcirculatory dysfunction in patients with circulatory shock after cardiovascular surgery: an observational study

**DOI:** 10.1038/s41598-022-19741-0

**Published:** 2022-09-10

**Authors:** John C. Greenwood, Fatima M. Talebi, David H. Jang, Audrey E. Spelde, Joseph E. Tonna, Jacob T. Gutsche, Jiri Horak, Michael A. Acker, Todd J. Kilbaugh, Frances S. Shofer, John G. T. Augoustides, Jan Bakker, Jacob S. Brenner, Vladimir R. Muzykantov, Benjamin S. Abella

**Affiliations:** 1 Department of Emergency Medicine, Center for Resuscitation Science, Perelman School of Medicine, University of Pennsylvania, Philadelphia, PA USA; 2 Department of Anesthesiology and Critical Care, Perelman School of Medicine, University of Pennsylvania, Philadelphia, PA USA; 3 Division of Cardiothoracic Surgery, Division of Emergency Medicine, University of Utah School of Medicine, Salt Lake City, USA; 4 Division of Cardiovascular Surgery, Department of Surgery, Perelman School of Medicine, University of Pennsylvania, Philadelphia, PA USA; 5 Department of Anesthesiology and Critical Care Medicine, Center for Mitochondrial and Epigenomic Medicine, The Children’s Hospital of Philadelphia, Philadelphia, PA 19104 USA; 6 Department of Epidemiology & Biostatistics, Department of Emergency Medicine Hospital, University of Pennsylvania, Philadelphia, PA USA; 7 Division of Pulmonary, Allergy, and Critical Care Medicine, New York University, New York, NY USA; 8 Department of Intensive Care Adults, Erasmus MC University Medical Center, Rotterdam, The Netherlands; 9 Division of Pulmonary, Allergy, & Critical Care, Perelman School of Medicine, University of Pennsylvania, Philadelphia, PA USA; 10 Department of Pharmacology and Center for Translational Targeted Therapeutics and Nanomedicine, Perelman School of Medicine, University of Pennsylvania, Philadelphia, PA USA; 11 Department of Emergency Medicine, Hospital of the University of Pennsylvania, 3400 Spruce Street, Philadelphia, PA 19104 USA

**Keywords:** Cardiovascular diseases, Diagnostic markers, Translational research

## Abstract

Persistent abnormalities in microcirculatory function are associated with poor clinical outcomes in patients with circulatory shock. We sought to identify patients with acutely reversible microcirculatory dysfunction using a low-dose topical nitroglycerin solution and handheld videomicroscopy during circulatory shock after cardiac surgery. Forty subjects were enrolled for the study, including 20 preoperative control and 20 post-operative patients with shock. To test whether microcirculatory dysfunction is acutely reversible during shock, the sublingual microcirculation was imaged with incident dark field microscopy before and after the application of 0.1 mL of a 1% nitroglycerin solution (1 mg/mL). Compared to the control group, patients with shock had a higher microcirculation heterogeneity index (MHI 0.33 vs. 0.12, *p* < 0.001) and a lower microvascular flow index (MFI 2.57 vs. 2.91, *p* < 0.001), total vessel density (TVD 22.47 vs. 25.90 mm/mm^2^, *p* = 0.005), proportion of perfused vessels (PPV 90.76 vs. 95.89%, *p* < 0.001*)* and perfused vessel density (PVD 20.44 vs. 24.81 mm/mm^2^, *p* < 0.001). After the nitroglycerin challenge, patients with shock had an increase in MFI (2.57 vs. 2.97, *p* < 0.001), TVD (22.47 vs. 27.51 mm/mm^2^, *p* < 0.009), PPV (90.76 vs. 95.91%, *p* < 0.001), PVD (20.44 vs. 26.41 mm/mm^2^, *p* < 0.001), venular RBC velocity (402.2 vs. 693.9 µm/s, *p* < 0.0004), and a decrease in MHI (0.33 vs. 0.04, *p* < 0.001. Thirteen of 20 patients showed a pharmacodynamic response, defined as an increase in PVD > 1.8 SD from shock baseline. Hemodynamics and vasoactive doses did not change during the 30-min study period. Our findings suggest a topical nitroglycerin challenge with handheld videomicroscopy can safely assess for localized recruitment of the microcirculatory blood flow in patients with circulatory shock and may be a useful test to identify nitroglycerin responsiveness.

## Introduction

Circulatory shock is a life-threatening, generalized form of acute circulatory failure where there is an imbalance between oxygen demand, supply, or utilization^1,2^. Traditionally shock is categorized by macrocirculatory etiologies including hypovolemic, distributive, obstructive, and cardiogenic shock, but downstream effects on functional capillary density and blood flow are heterogeneous^3^. A number of mechanisms have been identified for reduced microcirculatory blood flow during shock states, including inflammatory-mediated vascular endothelial injury, microthrombosis, and an inadequate balance between vasoconstrictive and vasodilating molecules which can lead to a global or heterogeneous reduction in capillary blood flow^4,5^. Unfortunately, many of the often used therapies to reverse hemodynamic derangements do not reverse microcirculatory impairment^6–8^. Interventions during the optimization and stabilization phases of shock resuscitation are limited, and often guided by surrogate markers of tissue perfusion^2^.

Nitric oxide (NO) impacts many of the key pathologic changes observed in shock, including regulation of vascular endothelial function, microvascular permeability, leukocyte adherence, and platelet activation at the capillary level^9^. Vascular endothelial injury and impaired endothelial NO production have been implicated as a mechanism of shock severity. Nitroglycerin (NTG) is an endothelium-independent nitric oxide donor and vasodilator that has been shown to increase capillary density and blood flow in patients with vasopressor-dependent septic shock and acute heart failure^10,11^. Initiation of vasodilatory therapy in patients with vasoactive-dependent shock may be counterintuitive and might only benefit a select group of patients. In this case, a strategy that identifies nitroglycerin-responsive patients prior to systemic administration would be ideal.

Microdosing novel therapeutics in conjunction with local tissue imaging has gained popularity in fields outside of critical care to test for important physiologic responses to treatment^12^. A microdose is defined as being below the dose calculated to yield a pharmacological effect and less than 100 µg of the active drug^13^. In healthy adults, low-dose topical nitroglycerin has been used to measure microcirculatory reserve using sublingual incident dark-field (IDF) microscopy, but this approach could also be used in patients with shock to test for a physiologic response without exposing patients to undesired effects that may be experienced with systemic administration^14^. Our primary aim was to determine if a microdose of topical nitroglycerin could improve microcirculatory density and capillary blood flow in critically ill patients with vasoactive-dependent circulatory shock. A secondary aim was to identify nitroglycerin responders using a patient-specific pharmacodynamic response threshold^15^.

## Results

Forty-four patients were consented for enrollment in the study. Twenty-four patients with post-operative shock received the topical nitroglycerin challenge and 20 preoperative patients served as a control group. Control patients had common chronic medical conditions for patients with cardiovascular disease but did not have any pre-operative end-organ injury such as chronic kidney disease, reduced left ventricular ejection fraction, chronic liver disease. Two patients were excluded from the experimental arm prior to receiving the study drug (one did not require post-operative vasopressors, and one did not have a properly functioning pulmonary artery catheter). Twenty-two total subjects received the study drug to assess for microcirculation responsiveness. Two additional subjects were excluded after enrollment for having an inadequate number of quality videos to meet consensus standards. The CONSORT flow diagram is included in Supplemental Data. Patient information and demographics are in Table 1. Perioperative and clinical data are in Table 2. There was no difference in clinical status between NTG responders and non-responders at any given time point.

**Table 1:** Patient demographics.

	Preoperative control n = 20	Nitroglycerin challenge n = 20
Age, years	67 ± 11	64 ± 9
Sex, male	15, 75%	15, 75%
euroSCORE II	1.6 ± 1.5	2.7 ± 2.9
**Performed operation (n, %)**		
Coronary artery bypass grafting (CABG)	7, 35%	8, 40%
CABG + valve replacement/repair	3, 15%	5, 25%
Valvular surgery only	7, 35%	4, 20%
Aortic surgery	3, 15%	3, 15%
**Comorbidities (n, %)**		
Hypertension	11, 55%	17, 85%
Diabetes	5, 25%	8, 40%
Heart failure with reduced ejection fraction (< 30%)	0, 0%	3, 15%
Chronic kidney disease	0, 0%	6, 30%

**Table 2:** Clinical variables of participants and by nitroglycerin responsiveness.

	Overall (N = 20)	NTG responders (N = 13)	NTG non-responders (N = 7)	*p*-value
**Perioperative details**				
Cardiopulmonary bypass time, min	117 ± 58	138 ± 53	79 ± 50	0.03
Cross-clamp time, min	83 ± 46	100 ± 46	55 ± 30	0.04
**Vasoactives**				
Epinephrine, n [mcg/kg/min]	16, 0.04 ± 0.02	10, 0.04 ± 0.02	6, 0.05 ± 0.03	ns
Norepinephrine, n [mcg/kg/min]	11, 0.07 ± 0.06	7, 0.08 ± 0.07	4, 0.06 ± 0.06	ns
Vasopressin, n [units/min]	3, 0.03 ± 0.06	1, 0.02	2, 0.04 [0.04–0.04]	ns
Phenylephrine, n [mcg/min]	2, 25 ± 0	1, 25 ± 0	1, 25 ± 0	ns
Milrinone, n [mcg/kg/min]	2, 0.125 ± 0	1, 0.125 ± 0	1, 0.125 ± 0	ns
vasopressor-inotrope score (VIS)	8.2 [2.7–11.7]	5.2 [2.2–12.1]	9.4 [5.7–10.9]	ns
**Laboratory values**				
Hemoglobin, mg/dL	12.0 ± 1.6	12.2 ± 1.4	11.6 ± 2.0	ns
Hematocrit, %	35.1 ± 4.4	35.0 ± 4.0	35.2 ± 5.6	ns
Lactate, mmol/dL	3.3 ± 1.8	3.0 ± 1.5	2.3 ± 1.2	ns
SvO2, %	69 ± 9	69 ± 9	68 ± 10	ns
PvaCO_2_, mmHg	5 ± 3	5 ± 3	4 ± 2	ns
Capillary refill time, sec	5 ± 1	5 ± 3	5 ± 3	ns
**Baseline post-operative hemodynamics**				
Left ventricular ejection fraction, %	54 ± 14	53 ± 8	56 ± 21	ns
Mean arterial pressure, mmHg	71 ± 6	71 ± 4	72 ± 9	ns
Central venous pressure, mmHg	9 ± 4	9 ± 4	10 ± 4	ns
Cardiac index, L/min/m^2^	2.8 ± 0.8	2.9 ± 1	3.0 ± 1	ns
SVR index, dynes ·s/cm^5^/m^2^	1835 ± 606	1850 ± 587	1806 ± 690	ns

Mean ± SD, Median [IQR].

Compared to the control group, patients with postoperative shock had reduced microvascular flow index (MFI), heterogeneity (MHI), total vessel density (TVD), proportion of perfused vessels (PPV), and perfused vessel density (PVD) (Table 3).

**Table 3:** Sublingual microcirculation of preoperative control subjects compared to patients with postoperative shock.

Microcirculation value	Preoperative control (n = 20)	Circulatory shock (n = 20)	*p*
MFI (AU)	2.91 ± 0.10	2.57 ± 0.26	*0.0001*
MHI (AU)	0.12 ± 0.15	0.33 ± 0.22	*0.0014*
TVD (mm/mm^2^)	25.90 ± 3.85	22.47 ± 3.47	*0.0052*
PPV (%)	95.89 ± 2.99	90.76 ± 5.42	*0.0007*
PVD (mm/mm^2^)	24.81 ± 3.51	20.44 ± 3.38	*0.0003*
RBCv (µm/s)	433.3 ± 153.1	402.2 ± 130.0	0.6138

Values are given as the mean ± SD.

Significant values are in italics.

### Video quality analysis

Three hundred and twenty-six video clips of the sublingual microcirculation were obtained, stabilized, and graded for quality prior to inclusion for final analysis. Eighty-six videos were excluded from the NTG challenge group for having a Massey score > 10, leaving 240 acceptable videos for final analysis. The analyzed video clips were of good quality with a mean Massey score of 0.3 ± 0.4 (illumination 0.0 ± 0.1, duration 0.0 ± 0.2, focus 0.1 ± 0.2, content 0.1 ± 0.2, stability 0.0 ± 0.1, pressure 0.1 ± 0.3). Bland–Altman bias was 0.021 (95% limit of agreement − 0.11 to 0.15).

### Effect of topical nitroglycerin on the sublingual microcirculation

Nitroglycerin administration reduced flow heterogeneity, increased microcirculatory density, and increased venular RBC velocity (RBCv) within 3 min of nitroglycerin microdosing (Table 4). There was no difference in total vessel density (TVD; 25.90 ± 3.85 vs. 27.51 ± 3.77 mm/mm^2^, *p* = 0.19), proportion of perfused vessels (PPV; 95.89 ± 2.99 vs. 95.91 ± 3.04%, *p* = 0.98), perfused vessel density (PVD; 24.81 ± 3.51 vs. 26.41 ± 3.50 mm/mm^2^, *p* = 0.16), microvascular flow index (MFI; 2.91 ± 0.12 vs. 2.97 ± 0.06, *p* = 0.26), and heterogeneity (MHI; 0.12 ± 0.15 vs. 0.04 ± 0.08, *p* = 0.15) between control subjects and patients with shock after the NTG challenge. All microcirculation parameters returned to baseline values 30 min after the NTG challenge.

**Table 4:** Microcirculation measurements during NTG challenge.

	Shock baseline	3-min post NTG	30-min post NTG	*p*
MFI (AU)	2.57 ± 0.26	2.97 ± 0.06	2.51 ± 0.34	< 0.0001_NTG_
MHI (AU)	0.33 ± 0.22	0.04 ± 0.08	0.44 ± 0.33	< 0.001_NTG_
TVD (mm/mm^2^)	22.47 ± 3.47	27.51 ± 3.77	24.46 ± 4.1	0.0009_NTG_; 0.04_post_
PPV (%)	90.76 ± 5.42	95.91 ± 3.04	89.08 ± 6.31	0.001_NTG_
PVD (mm/mm^2^)	20.44 ± 3.38	26.41 ± 3.50	21.88 ± 3.84	< 0.0001_NTG_
RBCv (µm/s)	402.2 ± 133.0	693.9 ± 250.0	376.8 ± 133.7	0.0004_NTG_

Values are given as the mean ± SD. One-way repeated measures ANOVA with Tukey correction performed compared to baseline assessment. Significant difference relative to baseline measurement for 3-min post NTG challenge reported as “NTG” and 30-min after NTG challenge as “post”.

**Figure 1:**
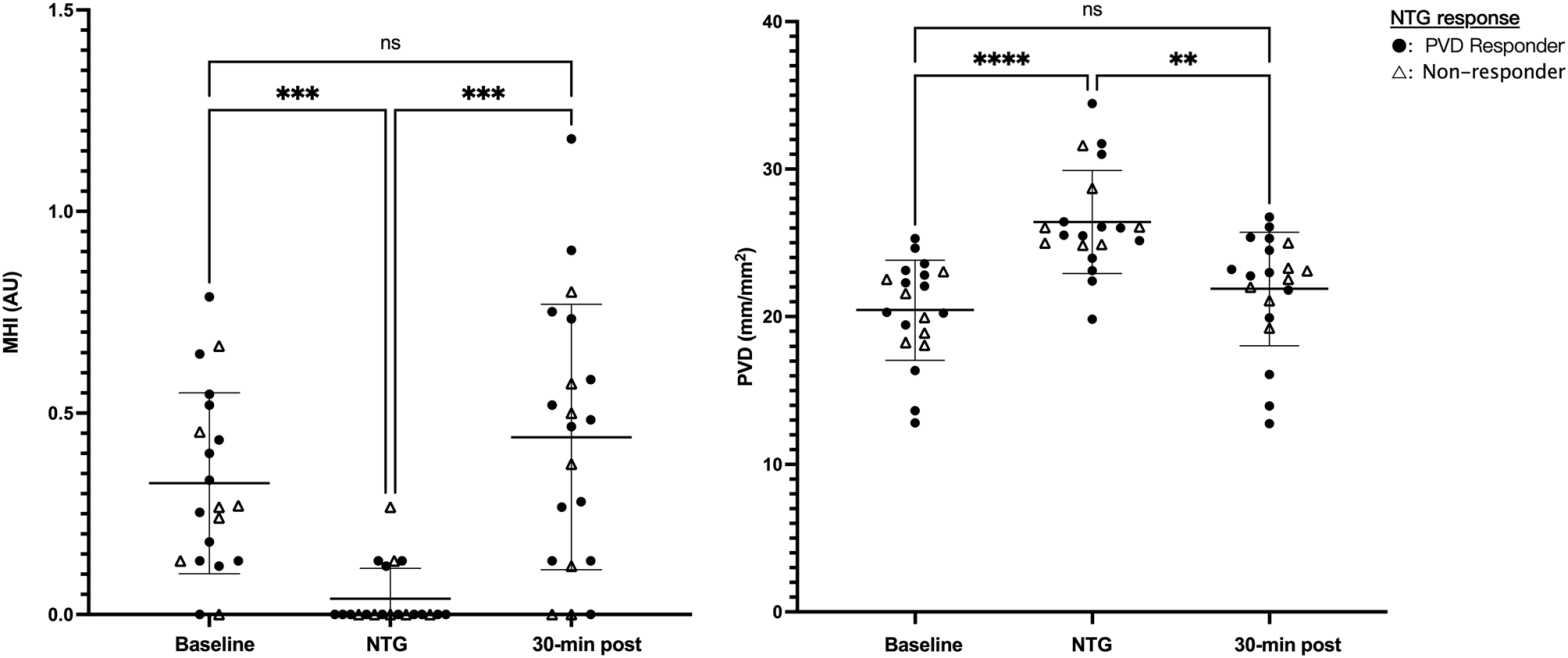
Individual response to the nitroglycerin (NTG) challenge. Thirteen out of 20 subjects experienced a PD response the topical nitroglycerin. Bars representing the mean with 95% CI. **p < 0.01, ***p < 0.001, ****p < 0.0001.

Pharmacodynamic (PD) response to nitroglycerin was defined as an increase in PVD > 5.46 mm/mm^2^ compared to baseline using a pooled baseline SD of 3.03. Baseline microcirculation characteristics of NTG responders and non-responders are listed in Table 5. Of the 20 subjects included in the final analysis, 13 had a significant PD response to the topical challenge (Fig. 1).

**Table 5:** Baseline microcirculatory function in NTG responders and non-responders.

	NTG responder baseline	NTG non-responder baseline	*p*
MFI (AU)	2.57 ± 0.24	2.57 ± 0.32	0.98
MHI (AU)	0.34 ± 0.22	0.31 ± 0.24	0.80
TVD (mm/mm^2^)	20.82 ± 3.08	25.53 ± 1.52	0.001
PPV (%)	91.34 ± 5.51	89.67 ± 5.50	0.53
PVD (mm/mm^2^)	19.13 ± 3.47	22.89 ± 1.20	0.013
RBCv (µm/s)	409.4 ± 141.7	388.9 ± 124.7	0.75

### Hemodynamics during the topical nitroglycerin challenge

**Figure 2:**
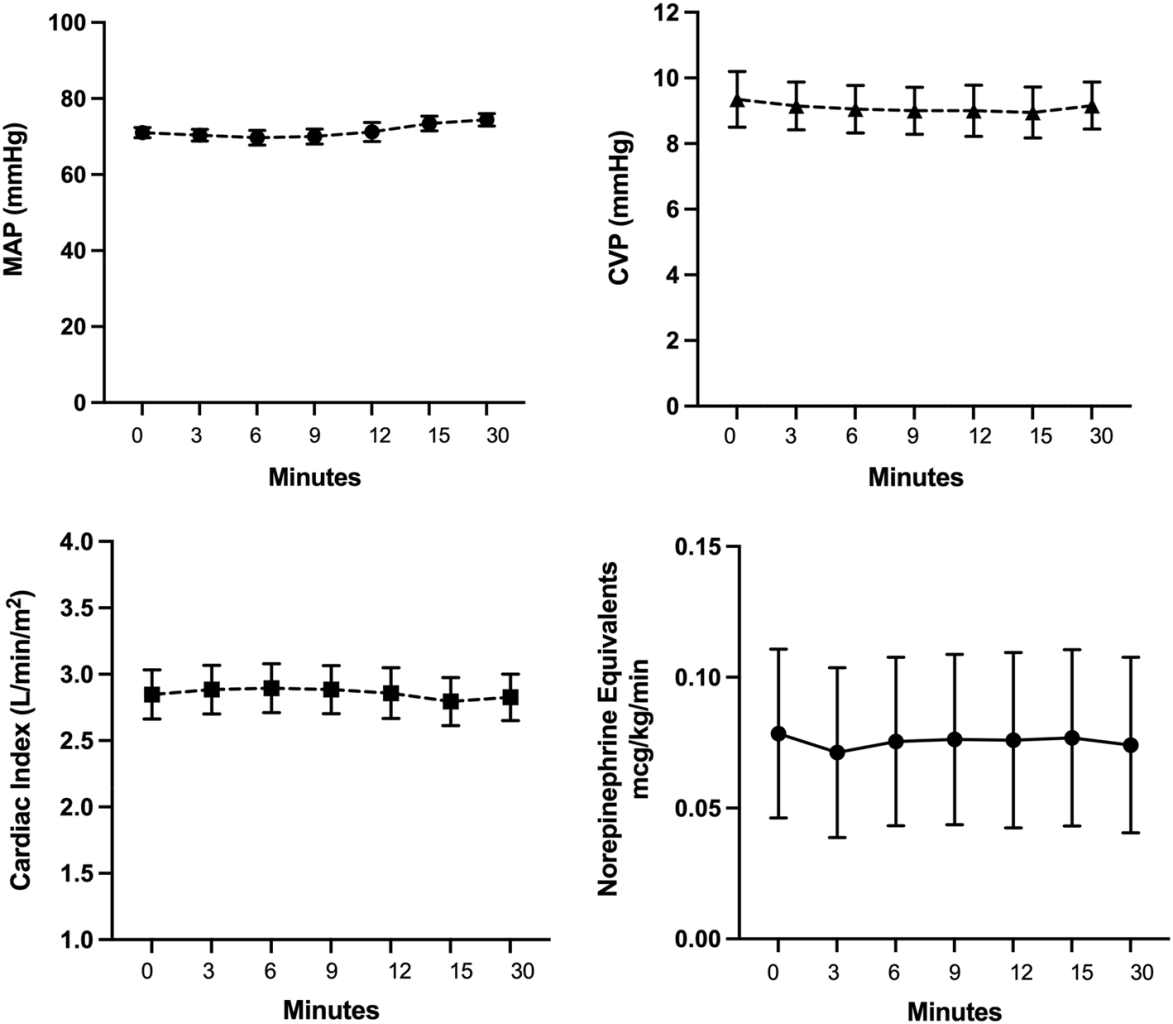
Hemodynamic response to NTG challenge (administration at “minute 0” timepoint). Datapoints represent the mean with SEM for mean arterial pressure (MAP), central venous pressure (CVP), cardiac index (CI), and vasopressor dose reported as norepinephrine equivalents.

There was no significant change in mean arterial blood pressure, central venous pressure, cardiac index, or systemic vascular resistance index during the 30-min challenge (Fig. 2). Patient norepinephrine equivalents at baseline, NTG administration, and at 30 min were mixed venous oxygen saturation and capillary refill time also did not change during the challenge.

## Discussion

Based on the results of this study, we were able to show that (i) sublingual capillary density and blood flow is reduced during postoperative circulatory shock compared to a preoperative control; (ii) reduced microcirculation density and flow could be increased using a topical endothelium-independent nitric oxide donor without any effect on systemic hemodynamics; and (iii) we could identify nitroglycerin responsive patients using a pharmacodynamic statistical method used in early phase 0 trials.

Circulatory shock is defined as an imbalance between oxygen demand, supply, or utilization^2^. Compared to our control group, patients with shock had lower capillary density and blood flow indicating reduced tissue perfusion. Current resuscitation strategies to reverse shock include augmenting intravascular volume and vasoactive therapies to normalize systemic hemodynamics, but unfortunately, these interventions do not always lead to improvements in capillary blood flow^7,16,17^. In this study, we found that topical nitroglycerin increased regional PVD and reduced heterogeneity in patients with shock which have been associated with poor clinical outcomes^7^. The MFI and RBCv of our shock cohort also increased after the NTG challenge, but both of these values appear to be close to values that may be considered within the normal range based on consensus definitions—specifically MFI^18^. Large studies to define human reference ranges have yet to be completed. It is unclear if targeting higher values would have any clinical or physiologic impact. The small vessel abnormalities identified with IDF are likely the result of a number of common post-cardiopulmonary bypass pathologies including a SIRS-related vasoplegia, low cardiac output, vascular endothelial injury, and microvascular shunting^5,19^. Nitric oxide donors are of particular interest as they have been found to be a mediator of endothelial ischemia–reperfusion injury and can provide a number of other tissue-protective effects including reduction of small vessel resistance, leukocyte adherence, platelet aggregation, and vascular endothelial cell permeability^20,21^.

The rapid change in perfused vessel density and flow heterogeneity suggests that an endothelium-independent nitric oxide donor can reverse early post-operative derangements in capillary blood flow. The time-course of the observed change in microcirculatory flow was consistent with the known pharmacokinetics of nitroglycerin. Although nitroglycerin does provide a vasodilatory effect on both arteries and veins, evidence suggest the most significant effect is on the venous system^22^. A significant reduction in venular pressure would increase the pressure differential across the capillary network to improve PVD and flow homogeneity identified with IDF. It is not possible to determine if the topical solution primarily acted on the pre- or post-capillary resistance vessels within the microvascular unit using IDF microscopy. As expected, the duration of the pharmacodynamic response was transient, lasting less than 30 min, which is consistent with well-studied nitroglycerin pharmacokinetics^23^.

Administration of a vasodilatory medication to patients with vasopressor-dependent hypotension is a significant departure from conventional treatment strategies and could result in significant hypotension. Topical nitroglycerin has been shown to improve local microcirculation without disturbing hemodynamics in hemorrhagic shock and in healthy volunteers^14,24^. We chose our dose of NTG based on the previous findings of Hilty et al., who also identified an increase in microcirculatory blood flow without a change in hemodynamics in healthy volunteers^14^. By incorporating handheld video microscopy (HVM) imaging, we were able to identify a pharmacodynamic effect of nitroglycerin using a solution that delivered less than 2% of the standard dose of sublingual nitroglycerin without causing a change in hemodynamics. Our method appears to be a low-risk test for identifying nitroglycerin responsiveness in patients with shock.

When evaluating our NTG response groups for potential confounders, both NTG responder and non-responder groups were similar. There was no difference in vasoactive medications, vasoactive dose, mean arterial pressure, central venous pressure, or other hemodynamic measures between NTG responders and non-responders. Nitroglycerin responders had a longer cardiopulmonary bypass and cross clamp time, which has been associated with more pronounced vascular endothelial injury and decreased NO production^5,25^. Our measurements of postoperative microcirculation parameters are similar to other reports in the literature when using consensus standard analysis methods^5,26^.

In our study, 65% of patients increased their PVD > 1.8 SD from baseline, indicating that NO donor responsiveness is likely individualized. In fact, NTG responders had lower baseline perfused vessel density, largely due to a reduced TVD. There was no difference in baseline MHI between groups, yet 15/20 subjects who received the NTG challenge resulted in an MHI that approached 0 indicating minimal flow heterogeneity and essentially uniform blood flow through most capillaries.

Previous research in vasoactive-dependent septic shock utilized a fixed-dose nitroglycerin infusion (30 mcg/min) which improved peripheral perfusion but did not improve sublingual microcirculatory function or survival compared to placebo^27^. It is possible that previous studies included patients that were either not responsive to nitroglycerin therapy or the dose administered was inadequate to stimulate regional blood flow in the vascular beds being imaged. The hesitancy to administer vasodilators in patients requiring vasopressor therapy is understandable, because current small molecule therapeutics cannot limit their effects to sites of microvascular injury. The development of novel NO-donor or NO-preserving therapeutics using nanocarrier technology, designed to target injured vascular endothelial cells, may be able to improve capillary blood flow in disturbed microvascular beds while minimizing undesirable side effects that can occur with current small molecule therapeutics^28–30^.

There are a few limitations to our study that should be identified. We did not perform a stepwise increase in nitroglycerin dosing, therefore it is possible that patients labeled as non-responders did not receive a high enough dose of nitroglycerin to improve capillary blood flow. While incorporating HVM to identify a real-time response to nitroglycerin can identify a physiologic response, significant time-constraints for the analysis of video sequences are a barrier to use in routine clinical practice. Development of software capable of providing real-time automated analysis would reduce the time required to interpret changes in PVD, allowing for more timely, individualized therapeutic interventions. Finally, there may be natural variation in sublingual microcirculation over time, which could account for a portion of the change we observed after the nitroglycerin challenge. While we did not perform an analysis to evaluate for regression to the mean, our findings overall reflected the anticipated change in microcirculatory function based on established nitroglycerin pharmacology. Future research that examines natural variation in microcirculatory flow over time on a larger dataset would be a valuable addition to the literature.

In conclusion, we were able to demonstrate that abnormalities in functional capillary density, heterogeneity, and flow during circulatory shock can be reversed using an endothelium-independent nitric oxide donor. The effect appears to be most pronounced in patients with abnormal perfused vessel density. Individual responsiveness can be safely tested with the use of a low-dose topical nitroglycerin solution in conjunction with HVM.

## Methods

### Patient selection

Adult patients (age > 18 years) receiving elective cardiac surgery requiring cardiopulmonary bypass were screened for eligibility from August to October 2021. Written informed consent was obtained from eligible patients prior to surgery. Patients were excluded if they had a nitroglycerin allergy, were taking oral phosphodiesterase inhibitors, were unable to tolerate microcirculatory flow image acquisition or did not have a pulmonary artery catheter for continuous cardiac output monitoring after surgery. Patients only received the study drug if they met the clinical criteria for circulatory shock, defined as having postoperative vasopressor-dependent hypotension or low cardiac output requiring inotropic support, with signs of end-organ injury (capillary refill time > 3 sec, lactate > 2 mmol/dL, SvO2 < 60%). A second cohort of patients were enrolled to serve as a preoperative control, which were matched to the experimental group for age, co-morbidities, and planned operation. Study data were collected and recorded using a well-established clinical database tool (REDCap, Vanderbilt University, Nashville, TN) hosted at the University of Pennsylvania^31,32^.

### Study design and drug administration

This investigator-initiated, open-label study was conducted at the Hospital of the University of Pennsylvania, an urban quaternary academic medical center. The study was approved by the University of Pennsylvania’s institutional review board, registered with ClinicalTrials.gov (NCT05102734), and conducted in accordance with the principles of the Declaration of Helsinki. Sample size was determined based on an anticipated 30% increase in TVD, and setting a one-sided α of 0.1, and ß of 0.8. A 1% topical nitroglycerin solution was prepared immediately prior to enrollment by reconstituting 400 mcg of nitroglycerin (Pfizer Pharmaceuticals, New York, NY, USA) in 4 mL of sterile water within a dropper bottle that would administer the solution in 0.05 mL aliquots. Two drops (0.1 mL) of the 1% nitroglycerin solution (5 mcg or 2.27 × 10^−2^ μmol per drop) were applied to the sublingual space after obtaining baseline post-operative microcirculation images. A microdose (approximately 1/50th of the lowest therapeutic sublingual dose) was administered to avoid any systemic effects.

### Microcirculatory imaging and analysis

Sublingual microcirculation imaging was performed using HVM (CytoCam, Braedius Medical BV, the Netherlands) in the preoperative staging area (for controls) or within 2 hours of arriving to the intensive care unit after surgery by a trained member of the investigative team (JCG or FMT)^33^. The sublingual microcirculation was measured at 3 time points: baseline, 3 min post-nitroglycerin, and then 30 min later. Images were obtained by gently placing the videomicroscope under the subject’s tongue until an adequate view of the microcirculation was obtained. A series of six second video clips (120 frames) duration were taken at each time point with attention to quality factors, especially the absence of pressure artifact, excess saliva, and proper location in accordance with the accepted consensus for microcirculation analysis. Image quality was assessed using the 6-factor Massey quality score, which scores each video for appropriate illumination, duration, focus, content, stability, and pressure. Images were only included in the final analysis if the Massey quality score was < 10^34^. Acceptable clips were deidentified and coded for analysis after enrollment was complete.

Microcirculation videos were exported using commercial software (CCTools 2, Braedius Medical BV, the Netherlands) and manually analyzed using the validated Automated Vascular Analysis software (AVA 3.2; Microvision Medical B.V., the Netherlands). Video analysis was performed by trained investigators (JCG and FMT) who were blinded to the conditions of the subject. Only microvessels ≤ 20 μm in diameter were included in the calculation of TVD and PVD.

Red blood cell flow velocity was calculated within appropriate venules 20–30 μm in diameter by manually measuring the slope of individual RBC movement within software generated space–time diagrams^18^. Microcirculatory function was quantified according to the current best practice guidelines for microcirculation imaging^18^. Blood flow within each microvessel was graded using a semiquantitative scale, based on the vascular flow pattern ranging from 0 to 3 (0 = no flow, 1 = intermittent, 2 = sluggish, or 3 = continuous flow). Vessel perfusion was dichotomized as nonperfused (no flow or intermittent flow) or perfused (continuous or sluggish) for the calculation of PPV and PVD.

### Individual pharmacodynamic response to the nitroglycerin challenge

An exploratory analysis was planned to identify the incidence of a patient specific pharmacodynamic response to the nitroglycerin challenge. A PD response threshold was set as a post-administration PVD > 1.8 standard deviations from the baseline, which was calculated by averaging the intra-patient variance between the three baseline PVD video sequences, which was then pooled across the entire cohort. This 1.8SD threshold was set to achieve 90 percent confidence interval (assuming a one-sided alpha, since there was only an anticipated increase in PVD). This method has been used in previous Phase 0 trials to provide a statistically rigorous threshold for individualized PD responses to novel therapeutic interventions^15^.

### Patient data and safety monitoring

Subject demographics, pre-operative risk score (euroSCORE II) and medical history were collected during initial screening. Cardiac index (CI), central venous pressure (CVP), pulmonary artery pressure (PAP), and mixed venous oxygen saturation (SvO_2_) were continuously monitored by pulmonary artery catheter (Edwards Lifesciences LLC, Irvine, CA, USA). Arterial blood pressure was measured continuously using a standard invasive arterial line. Systemic hemodynamic data, vasopressor infusion doses, and mixed venous oxygen saturation were recorded at baseline then every 3 min for 30 min after application of the topical nitroglycerin solution^35^. The vasopressor-inotrope score (VIS) was calculated to summarize the degree of post-operative vasoactive support was required at the time of the study^36^. Norepinephrine equivalents were calculated for each patient at each time point^37^.

### Statistical analysis

Data normality was assessed using the D’Agostino-Pearson omnibus normality test. Global hemodynamic and microcirculation variables are reported as mean ± SD. Variables that were not normally distributed are reported as median with interquartile range [25th–75th percentiles]. Student t-test was used to test for differences between normally distributed variables. Mann–Whitney U test was used to analyze non-normal data. Repeated measure one-way ANOVA with Tukey’s post-hoc testing was used to assess for changes in microcirculatory function during the nitroglycerin challenge. Inter-rater reliability between coders was assessed in 10% of the videos using a Bland–Altman plot. Statistical analysis was conducted using Prism v 8.0 (Graph-Pad Software, San Diego, CA). Statistical significance was assumed at *p* < 0.05.

### Ethics approval and consent to participate

This study was approved by the University of Pennsylvania’s institutional review board (IRB # 829765) and informed consent was obtained prior to enrollment. All consent forms were copied in triplicate, one given to the subject, the second placed in the official medical record, the third kept in a secured location within the PI’s office.

## Supplementary Information

Supplementary Information.

## Data Availability

All original data and materials are kept in a locally managed REDCap database at the University of Pennsylvania. The dataset supporting the results of this report is available via the Zenodo research data repository^38^. The deidentified microcirculation dataset can be found here: https://zenodo.org/record/5768720.

## Abbreviations

AU: Arbitrary units
CABG: Coronary artery bypass grafting
CI: Cardiac index
CVP: Central venous pressure
MAP: Mean arterial pressure
MFI: Microcirculatory flow index
MHI: Microcirculatory heterogeneity index
NO: Nitric oxide
NTG: Nitroglycerin
PAP: Pulmonary artery pressure
PPV: Proportion of perfused vessels
PVD: Perfused vessel density
RBCv: Red blood cell velocity
SvO2: Mixed venous oxygen saturation
TVD: Total vessel density
VIS: Vasopressor-inotrope score
