# Supplementary material for: Topical nitroglycerin to detect reversible microcirculatory dysfunction in patients with circulatory shock after cardiovascular surgery: an observational study
Source: Sci Rep. 2022 Sep 10;12:15257. doi: 10.1038/s41598-022-19741-0 (PMC9464203; doi:10.1038/s41598-022-19741-0)
Supplement: Supplementary file 1 — Supplementary Information. [file 41598_2022_19741_MOESM1_ESM.docx]

Assessed for eligibility (n=44)

Analyzed (n=20)

Analyzed (n=20)

- Excluded from analysis (n=2): Did not have an adequate number of videos that met quality standards for analysis.

Nitroglycerin challenge (n=22)

Nitroglycerin challenge subjects excluded prior to receiving study drug (n=2)

- No longer on vasopressors when study team arrived (n=1)
- PAC was not properly functioning (n=1)

## Enrollment

## Analysis

Healthy control (n=20)
